# Association of the mitochondrial regulator PGC-1α with diabetes mellitus and myocardial ischemia–reperfusion injury in coronary artery bypass grafting

**DOI:** 10.1186/s12872-026-06151-7

**Published:** 2026-06-19

**Authors:** Ayla Yildiz, Süleyman Yazici, Zülfiye Yildiz

**Affiliations:** 1 Department of Medical Biochemistry, Başakşehir Çam and Sakura City Hospital, Istanbul, Türkiye; 2 Cardiovascular Surgery Clinic, Başakşehir Çam and Sakura City Hospital, Istanbul, Türkiye; 3 Department of Anesthesiology and Intensive Care, Basaksehir Cam and Sakura City Hospital, İstanbul, Türkiye

**Keywords:** Diabetes mellitus, Ischemic heart disease, Myocardial ischemia-reperfusion injury, PPARγC1α

## Abstract

**Objectives:**

This study aimed to evaluate whether peroxisome proliferator-activated receptor gamma coactivator-1 alpha (PGC-1α) could serve as a marker of myocardial ischemia by examining its association with cross-clamp time, which reflects the duration of ischemia, in patients undergoing coronary artery bypass grafting (CABG).

**Methods:**

The study included 44 adult patients who underwent open-heart surgery at the Cardiovascular Surgery Clinic between June 2024 and December 2024. Serum samples separated from the blood collected after cross-clamping and at the pump outlet were analyzed in our hospital’s central laboratory for the PGC-1α study. PGC-1α levels were analyzed in terms of clinical parameters, lactate and high-sensitivity cardiac troponin T (hs-cTnT) levels of the patients.

**Results:**

Preoperative PGC-1α levels did not differ significantly from postoperative levels (2.06 ± 5.75 vs. 1.43 ± 3.35, *p* = 0.608), whereas hs-cTnT levels increased significantly after surgery (*p* < 0.001). Preoperative PGC-1α showed a moderate positive correlation with DM (*r* = 0.366, *p* = 0.015) but no significant association with other clinical variables, including ICU duration (*r* = 0.078, *p* = 0.616), glucose, or HbA1c (all *p* > 0.05). In the multivariable analysis, DM (odds ratio [OR] = 3.060, *p* < 0.05) and ICU duration (OR = 1.466, *p* < 0.05) were independently associated with PGC-1α, whereas preoperative glucose and HbA1c were not. ROC analysis demonstrated that hs-cTnT showed a significant discriminative ability for cross-clamp time in non-DM patients (AUC = 0.844, 95% CI: 0.684–1.000, *p* = 0.002), whereas this relationship was not observed in DM patients. In contrast, PGC-1α did not demonstrate a significant discriminative performance in either group (AUC = 0.300–0.535, *p* > 0.05).

**Conclusion:**

PGC-1α was not associated with cross-clamp time or conventional markers of acute myocardial injury in patients undergoing coronary artery bypass grafting, indicating that it does not reflect ischemic duration. In contrast, hs-cTnT levels were significantly associated with the cross-clamp time in non-diabetic patients, whereas this relationship was not observed in diabetic individuals. The association of PGC-1α with diabetes mellitus and ICU duration suggests that it may reflect metabolic status rather than acute myocardial injury.

**Supplementary Information:**

The online version contains supplementary material available at 10.1186/s12872-026-06151-7.

## Introduction

Myocardial ischemic injury is a medical condition that occurs after impairment of the coronary artery supply and clinical syndromes [1]. Ischemic heart disease is associated with high mortality and morbidity worldwide and may lead to arrhythmia, cardiac dysfunction, myocardial infarction, and sudden death; however, its underlying mechanisms and biochemical processes remain complex and not fully understood [2–4]. Restoration of blood flow after myocardial ischemia causes serious dysfunction known as myocardial ischemia-reperfusion injury (MIRI). There is no effective treatment method for MIRI, which is characterized by cardiomyocyte death after ischemia in the heart muscles [5, 6]. Although there is a focus on preventing ischemic injury or reducing its effects for preventive treatment or precaution, treatment alternatives for the level of perfusion injury are relatively limited [7–9]. However, to understand the clinical picture and prevent MIRI progression, the level of myocardial injury or its biochemical processes must be predicted.

Peroxisome proliferator-activated receptor gamma coactivator-1 alpha (PGC-1α), a transcriptional coactivator abundantly expressed in the heart, plays a central role in regulating mitochondrial biogenesis and function, thereby maintaining cellular energy homeostasis [10–13]. It plays a key role in cardiac energy metabolism and ATP production and supports myocardial function under physiological conditions [14]. During myocardial ischemia–reperfusion, these processes are disrupted, leading to impaired energy balance and cellular dysfunction [15, 16]. In this context, evaluating PGC-1α levels may provide insights into the metabolic alterations associated with myocardial ischemia-reperfusion injury.

Although several studies have investigated myocardial ischemia–reperfusion injury (MIRI), there remains a lack of comprehensive data elucidating its biochemical mechanisms and identifying reliable biomarkers. Therefore, there is a need to better understand the underlying metabolic processes and potential indicators of ischemic burden. This study aimed to evaluate whether PGC-1α could serve as a marker of myocardial ischemia by examining its association with cross-clamp time, which reflects the duration of ischemia, in patients undergoing coronary artery bypass grafting (CABG).

## Methods

### Research model

In our study, we planned to collect samples for PGC-1α in a red-capped gel-filled vacuum tube (serum) from 44 patients who applied to the Cardiovascular Surgery Clinic of Başakşehir Çam and Sakura City Hospital and were scheduled to undergo open heart surgery. Blood samples were collected via retrograde cardioplegia cannula at two distinct time points: before aortic cross-clamping and immediately following cross-clamp removal. This study was designed as an observational longitudinal investigation grounded in a relational (correlational) research framework.

### Patients

This study included 44 adult patients who underwent open-heart surgery at the Cardiovascular Surgery Clinic between June 2024 and December 2024. The inclusion criteria were as follows.

- Patients over 18 years of age
- who have undergone open heart surgery

The exclusion criteria were as follows.

- Patients with renal failure
- Patients with decompensated heart failure
- Patients receiving inotropic support or IABP support under emergency conditions
- Patients who developed postoperative conditions known to independently elevate lactate levels (e.g., low cardiac output syndrome, severe anemia, mesenteric ischemia, septic shock, renal failure) were excluded to avoid confounding factors in the assessment of tissue hypoperfusion.

### Procedures

The patients’ routine blood gas (lactate) parameter data were obtained using HBYS. Serum samples separated from the blood collected before and after aortic cross-clamping were stored in a -80 °C deep freezer at our hospital’s central laboratory for the PGC-1α study. Serum samples were transferred into flat-capped gel tubes and centrifuged at 1,500 rpm/min for 10 min. The supernatant was collected in 1 ml wells and frozen at -80◦C as soon as possible. A commercially available enzyme-linked immunosorbent assay (ELISA) kit (Elabscience, Catalog No: E-EL-H1359) was used to measure serum PGC-1α concentrations according to the manufacturer’s instructions, with a sensitivity of 0.1 ng/mL, a detection range of 0.16–10 (ng/mL) and an intra-assay coefficient of variation < 10%. Samples with pre-analytical issues (e.g., hemolysis, lipemia, clotting, or insufficient volume) were excluded prior to the analysis; therefore, no missing data were present in the dataset. Blood samples were collected via retrograde cardioplegia cannula before aortic cross-clamping and after cross-clamp removal, representing the ischemic and reperfusion phases, respectively. hs-cTnT (ng/L) was included as a well-established biomarker of myocardial injury. Lactate levels were assessed as indicators of tissue hypoperfusion and metabolic stress during cardiopulmonary bypass.

All parameters were studied by the researchers at our hospital’s medical biochemistry laboratory using existing laboratory equipment.

This study included patients who underwent coronary artery bypass surgery (CABG). Our Cardiovascular Surgery clinic’s standard routine anesthesia protocol was applied to all cases as follows:

- As part of preoperative preparation, oral antidiabetic medications were discontinued under the supervision of endocrinologists, and insulin therapy was initiated. Antihypertensive medications, statins, and other ancillary medications were discontinued prior to the surgery. Patients with comorbidities other than diabetes and hypertension were excluded from the study. All these procedures represent standard methods routinely applied in cardiac surgery.
- A standardized protocol was applied for anesthesia induction in all patients. Midazolam, thiopental (pentothal), rocuronium, and fentanyl were used in all cases for anesthesia induction and maintenance; sevoflurane was the preferred inhalational anesthetic agent. Standard blood cardioplegia containing potassium chloride and magnesium sulfate was administered anterograde and retrograde, respectively. No adjuvant was added to the cardiopulmonary solution.

### Statistical methods

In this study, nominal data, such as sex and comorbidity, were defined by frequency analysis. Measurement parameters were defined as mean, standard deviation, median, and range. Kolmogorov Smirnov test was performed for normality distribution of measurement data. Since data distributions did not comply with the standard normal distribution, Spearman’s rho correlation and Generalized Linear Model (Logit) analyses were used in correlation analyses and regression analyses due to linearization deviations [17, 18]. All analyses were performed in SPSS 25.0 for Windows program, with 95% Confidence Interval and 0.05 significance level. A priori power analysis for a paired-samples t-test (two-tailed, α = 0.05, effect size dz = 0.5) indicated that a minimum sample size of 34 participants was required to achieve 80% statistical power.

## Results

A total of 44 patients, including 16 females (36.4%) and 28 males (63.6%), were included in the study. Baseline demographic, clinical, laboratory, and operative characteristics of the study population are summarized in (Table 1).

**Table 1:** Baseline characteristics of patients and descriptive statistics

	Mean ± SD	Median (Min-Max)
Age, year	57.29 ± 10.02	58.00 (20.00–76.00)
BMI, kg/m^2^	28.39 ± 5.11	28.10 (17.09–43.28)
Pump time, minutes	141.16 ± 36.11	143.50 (44.00-229.00)
Cross-clamp time, minutes	105.07 ± 27.27	103.50 (52.00-172.00)
ICU duration, days	2.18 ± 0.75	2.00 (1.00–5.00)
Hospital duration, days	10.52 ± 9.02	8.00 (5.00–49.00)
Postop extubation duration, days	10.32 ± 3.76	10.00 (3.00–24.00)
IABP	0.17 ± 1.09	0.00 (0.00–7.00)
EF	49.94 ± 11.88	55.00 (8.00–60.00)
Number of Cardioplegia	3.34 ± 0.77	3.00 (2.00–5.00)
Preop lactate (mmol/L)	1.94 ± 1.07	1.75 (0.50–5.50)
Postop lactate(mmol/L)	4.06 ± 1.30	3.80 (1.60-9.00)
Lactate 10th min(mmol/L)	4.00 ± 4.54	3.05 (1.00–28.00)
Postop EF	51.05 ± 9.87	55.00 (25.00–60.00)
Preop glucose (mg/dL)	120.84 ± 26.40	112.00 (90.00-202.00)
HbA1c, %	6.44 ± 1.62	5.80 (4.80–11.40)

*BMI *Body Mass Index, *PGC-1α *Peroxisome proliferator-activated receptor gamma coactivator-1α, *ICU *Intensive Care Unit, *IABP *Intraaortic Balloon Pumping, *EF *Ejection Fraction

PGC-1α level decreased after the operation, with an insignificant difference (*p* > 0.05). The hs-cTnT levels of the patients significantly increased after the operation (*p* < 0.05) (Table 2).

**Table 2:** Preop and postop PGC-1α, hs-cTnT and lactate changes of patients

	Mean ± SD	Median (Min-Max)	*p* value
PGC-1α preop	2.06 ± 5.75	0.26 (0.02–27.79)	0.608^a^
PGC-1α postop	1.43 ± 3.35	0.29 (0.04–19.09)	
hs-cTnT preop	26.95 ± 51.72	12.60 (3.00-345.00)	0.000^a^
hs-cTnT postop	907.60 ± 1316.30	564.00 (35.80–7099.00)	
Lactate preop	1.87 ± 1.03	1.70(0.50–5.50)	< 0.001^a^
Lactate postop	4.06 ± 1.29	3.80(1.60-9.00)	

a. Wilcoxon Signed Rank Test

*SD *Standard Deviation, *hs-cTnT *High-Sensitivity Cardiac Troponin T

Spearman’s rho correlation analysis revealed a significant association between preoperative PGC-1α levels and diabetes mellitus; however, no significant correlations were identified with the other clinical, laboratory, or operative variables evaluated in the study (Table 3).

**Table 3:** Spearman’s rho correlation analysis results for correlations between preop PGC-1α and clinical parameters

PGC-1α preop	*r*	*p*
Pump time, minutes	0.196	0.201
Cross-clamp time, minutes	0.158	0.307
ICU duration, days	0.078	0.616
Hospital duration, days	0.179	0.245
Postop extubation duration, days	0.068	0.660
IABP	0.201	0.209
EF	0.113	0.465
Cardioplegia number	0.168	0.277
Preop lactate(mmol/L)	0.114	0.459
Postop lactate(mmol/L)	-0.181	0.251
Lactate 10th min(mmol/L)	-0.142	0.360
hs-cTnT preop(ng/L)	-0.156	0.312
hs-cTnT ICU discharge(ng/L)	0.132	0.392
hs-cTnT 24 h(ng/L)	-0.036	0.815
Postop EF	0.004	0.982
HT	0.025	0.872
DM	0.366^*^	0.015
Smoking	-0.157	0.308
Mortality	0.138	0.371
Age, year	0.050	0.747
BMI, kg/m^2^	0.098	0.525
HbA1c, %	0.289	0.057

*BMI* Body Mass Index, *PGC-1α *Peroxisome proliferator-activated receptor gamma coactivator-1α, *ICU *Intensive Care Unit, *IABP *Intraaortic Balloon Pumping, *EF *Ejection Fraction, *CK-MB *Creatine Kinase MB, *hs-cTnT *High-Sensitivity Cardiac Troponin T

**Fig. 1:**
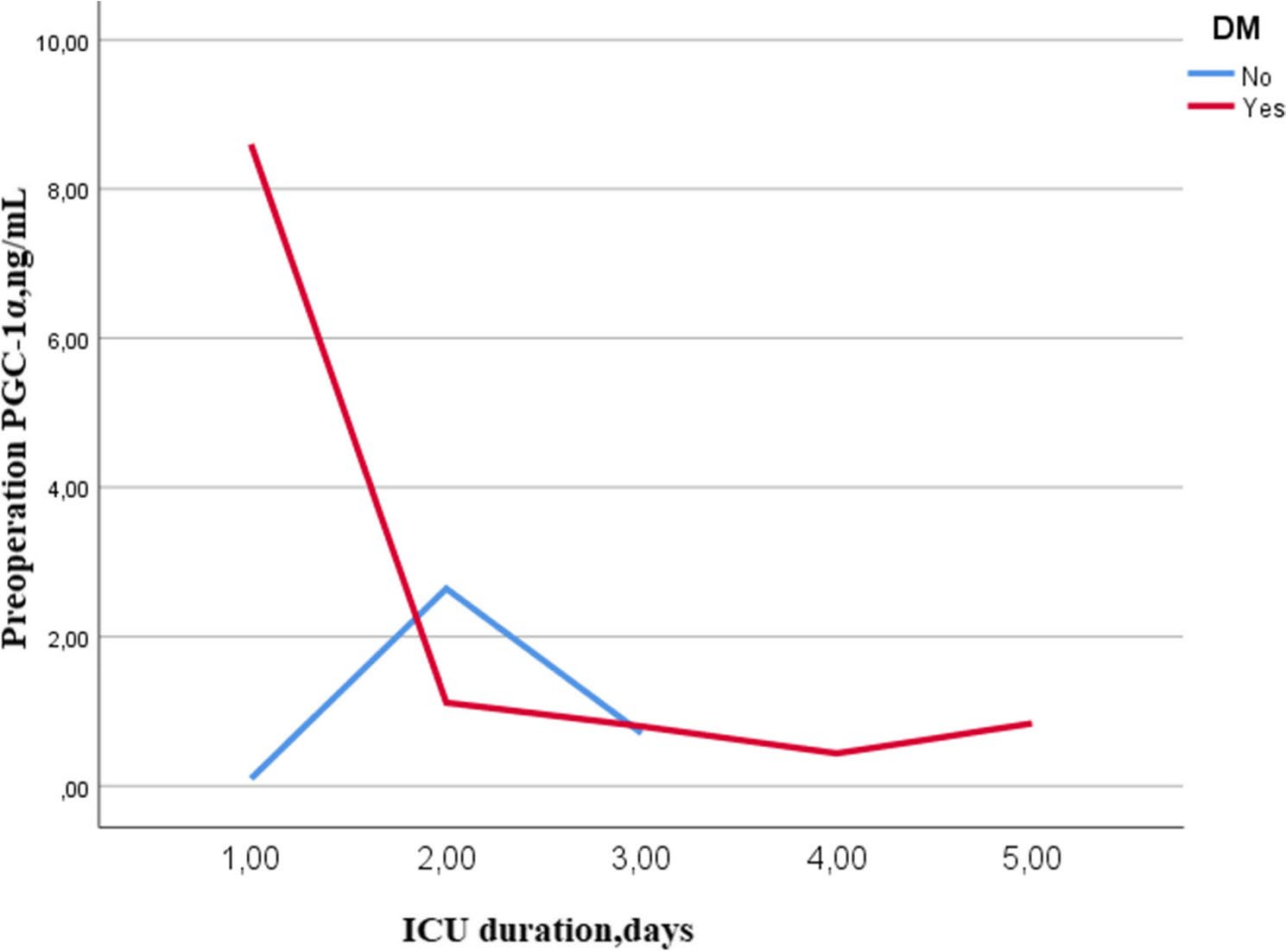
Preoperative PGC-1α and ICU duration change according to DM comorbidity

The mean preoperative PGC-1α level in patients with DM (2.05 ± 4.25) was lower than that in patients without DM (2.07 ± 6.39). In DM patients, there was a negative trend between preoperative PGC-1α and ICU duration (Fig. 1).

**Fig. 2:**
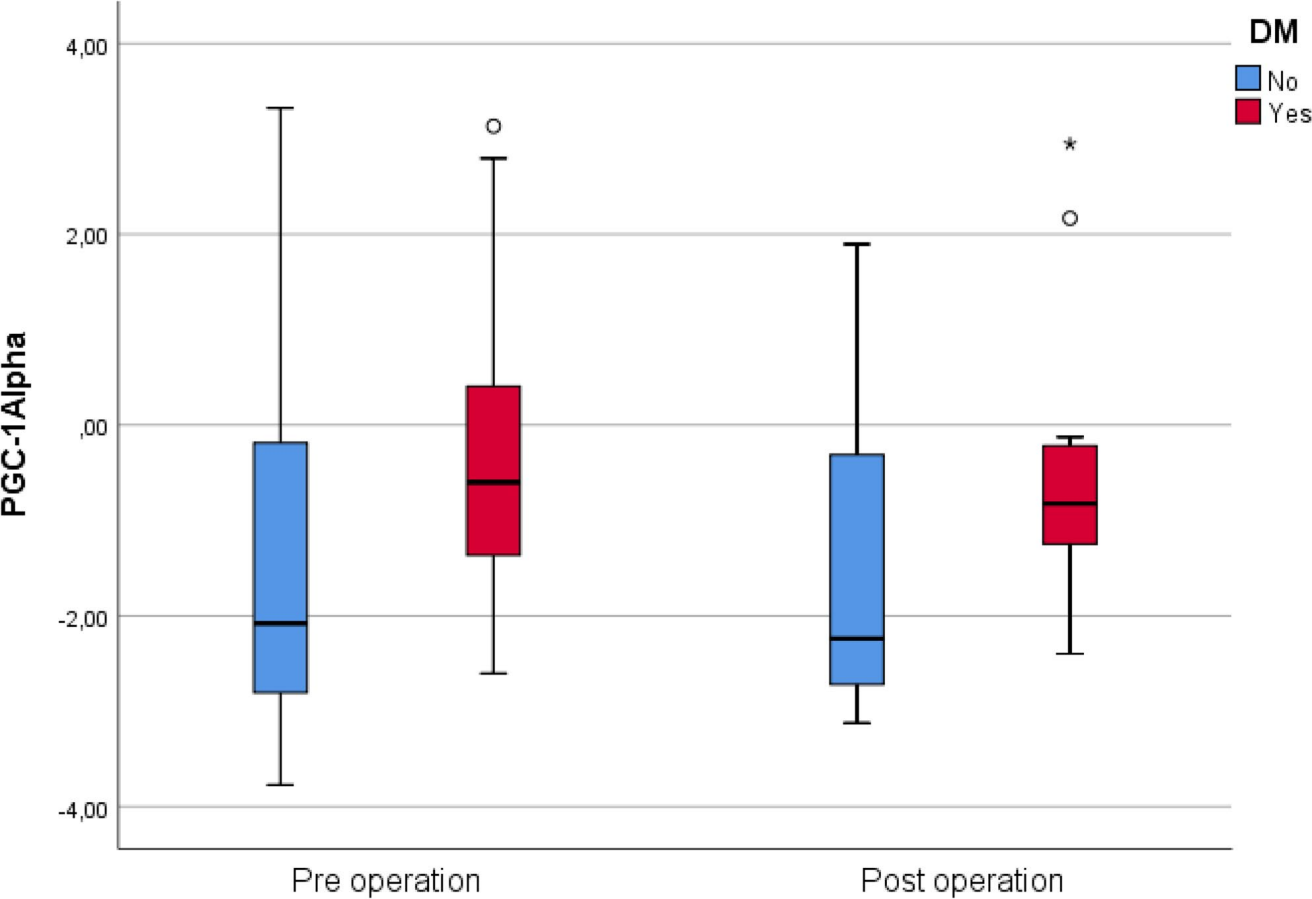
PGC-1α levels of DM patients before and after operation

PGC-1α levels of DM patients before and after operation were lower than non-DM patients, and DM patients had lower PGC-1α ranges (Fig. 2).

**Fig. 3:**
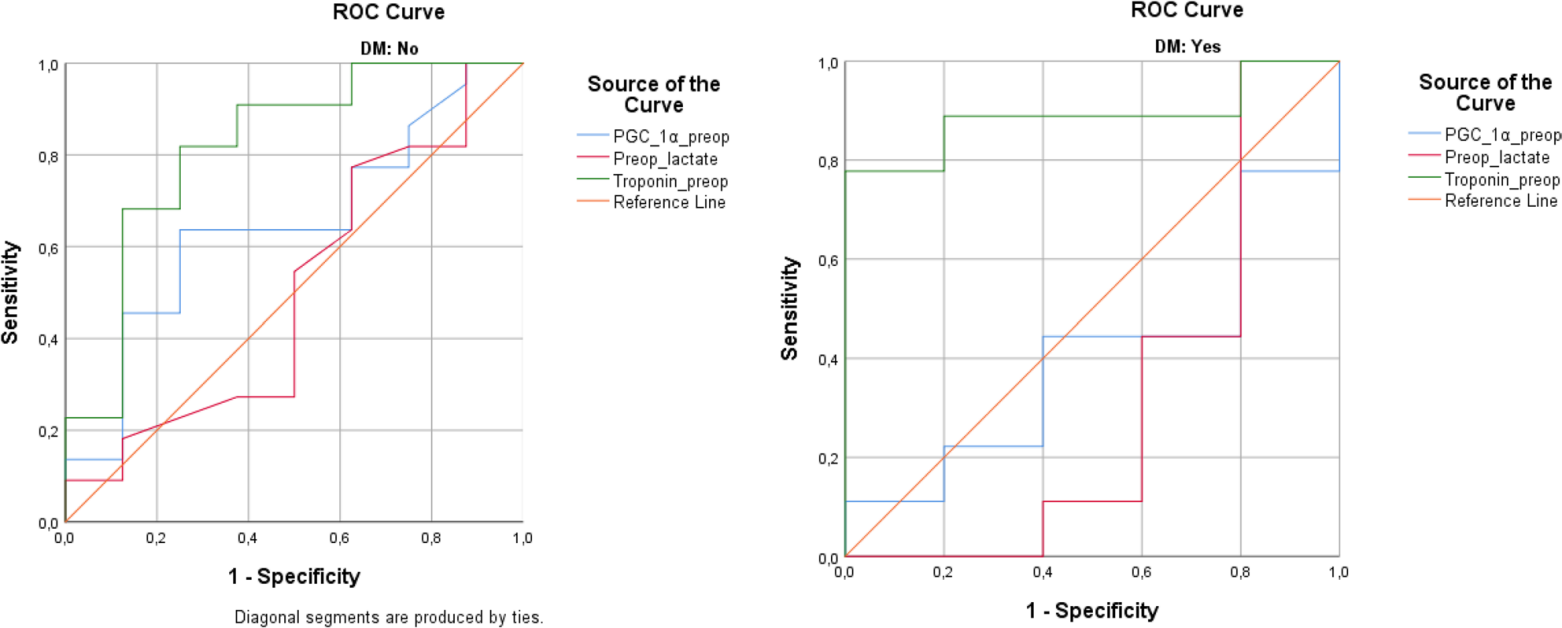
ROC curve analysis for the association between biomarkers and cross-clamp time in DM and non-DM patients

ROC analysis demonstrated that hs-cTnT was not significantly associated with cross-clamp time in patients with diabetes mellitus (*p* > 0.05), whereas a significant association was observed in non-diabetic patients (Fig. 3).

PGC-1α was not significantly associated with cross-clamp time in either DM or non-DM patients (*p* > 0.05). Similarly, hs-cTnT was not significantly prognostically associated with cross-clamp time in patients with DM (Table 4).

**Table 4:** ROC curve analysis results for associations with cross-clamp time in DM and non-DM patients

Test Result Variable(s)	Area	Std. Error	*p*	Asymptotic 95% Confidence Interval	
				Lower Bound	Upper Bound
*Non-DM patients*					
PGC-1α preop(ng/mL)	0.535	0.108	0.751	0.323	0.747
Preop lactate(mmol/L)	0.374	0.114	0.250	0.152	0.597
hs-cTnT preop (ng/L)	0.844	0.082	0.002	0.684	1.000
*DM patients*					
PGC-1α preop(ng/mL)	0.300	0.184	0.390	0.000	0.661
Preop lactate(mmol/L)	0.800	0.170	0.197	0.466	1.000
hs-cTnT preop(ng/L)	0.750	0.136	0.283	0.483	1.000

The effects of BMI, preop glucose, HbA1c, and preop PGC-1α on cross-clamp time were statistically insignificant for both non-DM and DM patients (Table 5).

**Table 5:** Generalized linear model (logit) for effects for BMI, preop glucose and preop PGC_1α on cross-clamp time for non-DM and DM patients

Parameter	B	Std. Error	95% Wald Confidence Interval		Hypothesis Test		
			Lower	Upper	Wald X^2^	df	*p*
*Non-DM patients*							
BMI, kg/m^2^	1.447	1.095	-0.699	3.593	1.746	1	0.186
Preop glucose(mg/dL)	-0.745	0.454	-1.635	0.146	2.688	1	0.101
HbA1c, %	3.966	14.328	-24.116	32.049	0.077	1	0.782
PGC-1α preop(ng/mL)	0.293	0.6985	-1.076	1.662	0.176	1	0.675
*DM patients*							
BMI, kg/m^2^	-2.185	1.244	-4.623	0.253	3.087	1	0.079
Preop glucose(mg/dL)	-0.480	0.328	-1.124	0.163	2.138	1	0.144
HbA1c, %	-2.163	4.700	-11.375	7.050	0.212	1	0.645
PGC-1α preop(ng/mL)	-1.338	1.850	-4.964	2.287	0.524	1	0.469

Effects of BMI, preop glucose, HbA1c and postop PGC- 1α on cross-clamp time were statistically insignificant for both non-DM and DM patients (Table 6).

**Table 6:** Generalized linear model (logit) for effects for BMI, preop glucose and postop PGC_1α on cross-clamp time for non-DM and DM patients

Parameter	B	Std. Error	95% Wald Confidence Interval		Hypothesis Test		
			Lower	Upper	Wald X^2^	df	*p*
*Non-DM patients*							
BMI, kg/m^2^	1.657	1.012	-0.327	3.641	2.678	1	0.102
Preop glucose(mg/dL)	-0.727	0.455	-1.619	0.166	2.546	1	0.111
HbA1c,%	5.655	14.701	-23.158	34.468	0.148	1	0.700
PGC-1α postop(ng/mL)	-0.147	1.169	-2.438	2.144	0.016	1	0.900
*DM patients*							
BMI, kg/m^2^	-2.112	1.176	-4.417	0.193	3.224	1	0.073
Preop glucose(mg/dL)	-0.464	0.312	-1.075	0.148	2.205	1	0.138
HbA1c, %	-2.219	4.415	-10.873	6.435	0.252	1	0.615
PGC-1α postop(ng/mL)	-3.859	2.718	-9.185	1.467	2.016	1	0.156

## Discussion

In this study, we explored whether PGC-1α could reflect myocardial ischemia by analyzing its relationship with cross-clamp time, a surrogate marker of ischemic duration, in patients undergoing coronary artery bypass grafting (CABG).

Determining the level of myocardial injury in MIRI cases and identifying the factors affecting it may significantly contribute to the treatment of patients and reduce mortality rates. Wang et al. [19] showed in their studies that PGC-1α activation, which increases with exercise, is inversely proportional to the level of myocardial injury. Hua et al. [20] reported that PGC-1α levels are associated with myocardial ischemia reperfusion in diabetic rats. Hua et al. reported that PGC-1α levels induced by metformin are associated with myocardial ischemia-reperfusion. Tian et al. [21] reported that PGC-1α levels are inversely related to myocardial ischemia reperfusion levels in rats treated with Tilianin. Zhu et al. [22] showed that PGC-1α levels may be an indicator of myocardial ischemia reperfusion levels in diabetic rats. Li et al. [23] reported in their studies that PGC-1α levels may be an indicator of the level of myocardial ischemia reperfusion. Similarly, Lu et al. [24] and Zhang et al. [25] reported in their studies that PGC-1α levels are inversely related to the level of myocardial ischemia reperfusion. In these studies, PGC-1α stood out for providing the energy needs of the heart in the form of ATP.

In this study, no statistically significant difference was observed between the preoperative and postoperative PGC-1α levels (*p* = 0.608), whereas hs-cTnT and lactate levels showed marked and statistically significant increases following surgery (both *p* < 0.001). These findings suggest that PGC-1α is more closely related to metabolic and mitochondrial processes rather than serving as a biomarker of acute myocardial injury. Recent studies have also explored alternative biomarkers in cardiovascular diseases, highlighting the need for more reliable indicators than conventional markers [26]. One of the most extensively studied biomarkers, hs-cTnT, is strongly associated with myocardial injury and clinical outcomes, particularly after coronary artery bypass grafting [27]. Similarly, lactate levels have been reported to reflect tissue hypoperfusion and are associated with clinical outcomes, such as intensive care unit duration and mortality [28]. In contrast, PGC-1α plays a central role in mitochondrial biogenesis and cellular energy metabolism and is mainly associated with adaptive cellular responses rather than acute myocardial injury [29, 30]. In this context, the present findings support the role of hs-cTnT and lactate as reliable indicators of acute myocardial injury and perfusion disturbances, while suggesting that PGC-1α may primarily reflect metabolic status rather than acute ischemic injury.

In addition, the preoperative PGC-1α level was significantly correlated with DM. The correlations between PGC-1α and other clinical parameters were statistically insignificant. PGC-1α levels were not associated with surgical parameters or conventional cardiac injury markers such as hs-cTnT and lactate. This may be explained by the fact that PGC-1α primarily reflects mitochondrial and metabolic regulation rather than acute myocardial injury [29, 30], whereas hs-cTnT is a well-established biomarker that is directly released in response to cardiomyocyte damage [27], and lactate levels reflect tissue hypoperfusion [31].

Indeed, routine hs-cTnT levels were evaluated at three distinct time points—preoperatively, at ICU discharge, and 24 h postoperatively—showing no significant correlation with PGC-1α. Consequently, these findings should be interpreted within the context of the available data points, as serial measurements were not performed to monitor the continuous dynamic course of PGC-1α.

To the best of our knowledge, no previous study has directly evaluated the relationship between PGC-1α levels and cross-clamp time. The lack of association observed in our study may reflect the distinct nature of these variables, as cross-clamp time is largely determined by surgical complexity and procedural factors, whereas PGC-1α is related to biochemical and metabolic pathways [29]. In addition, PGC-1α may be more closely linked to mitochondrial adaptive responses and oxidative stress during the reperfusion phase than during the acute duration of ischemia [32]. Furthermore, serum PGC-1α levels may not fully reflect myocardial tissue activity, which could have contributed to the lack of an observed association. Finally, the relatively small sample size may have limited the statistical power to detect moderate associations, particularly for secondary outcomes. No significant collinearity was observed among the variables included in the regression models.

Moreover, the mean preoperative PGC-1α level was lower in patients with diabetes mellitus than in those without diabetes mellitus. In addition, a negative trend was observed between preoperative PGC-1α levels and intensive care unit (ICU) duration in the diabetic subgroup.

The association between PGC-1α and ICU length of stay may be explained by its regulatory function in mitochondrial activity and cellular stress responses. Ischemia–reperfusion injury during cardiopulmonary bypass is characterized by mitochondrial dysfunction and increased production of reactive oxygen species (ROS) [33, 34]. In this context, PGC-1α may exert a protective effect by regulating mitochondrial antioxidant systems and limiting oxidative stress–induced myocardial injury [35]. Accordingly, higher preoperative PGC-1α levels may contribute to reduced myocardial damage, fewer postoperative complications, and shorter ICU stay.

In addition, the lack of association between preoperative glucose and HbA1c levels and PGC-1α in our study may be explained by the fact that PGC-1α is primarily regulated by acute metabolic and mitochondrial stress responses rather than by chronic glycemic status. Additionally, perioperative metabolic alterations related to surgical stress may have further obscured this relationship. These findings are consistent with studies demonstrating that PGC-1α regulation is mainly driven by mitochondrial and metabolic signaling pathways [11]. These findings are supported by human studies showing that even early metabolic disturbances, such as prediabetes [36], are associated with impaired cardiac function, highlighting the link between metabolic dysregulation and cardiac performance.

Mahmood et al. [37] evaluated PGC-1α expression in right atrial tissue samples before and after cardiopulmonary bypass. In contrast, the present study measured serum PGC-1α levels, and therefore, important methodological differences exist with respect to both the biological specimen and the assessment approach. In our study preoperative PGC-1α levels were lower in the DM group; however, the difference was too small to suggest a meaningful clinical or biological effect.

In this study, a separate healthy control group was not included, as the design primarily focused on perioperative myocardial ischemia–reperfusion dynamics. Moreover, because ischemia–reperfusion can only be evaluated under surgical conditions, assessing this process in healthy individuals is not methodologically appropriate. Nevertheless, previously published data provide a reference for physiological PGC-1α levels. In the study by Chen et al. [38], the mean serum PGC-1α concentration in healthy individuals was reported as 2.01 ng/mL. This value was found to be highly similar to the mean preoperative PGC-1α level observed in our patient cohort (≈ 2.06 ng/mL; *p* = 0.95 ) Suplement Fig. 1.

### Limitations of the study

This study had several important limitations. Although prospectively designed, its observational nature precludes causal inferences, meaning the results should be interpreted as associative. Additionally, because the study was conducted at a single center, variations such as different surgical techniques used in other institutions could not be evaluated, potentially limiting the external applicability of the findings. Nonetheless, the fact that all surgeries were performed by the same surgical team ensured a highly standardized technical approach, minimizing procedural confounding factors.

Due to the limited number of patients with DM in our cohort, subgroup analyses (e.g., insulin-dependent vs. non-insulin-dependent DM) were not feasible, restricting the evaluation of potential heterogeneity within the diabetic population. This limitation may also have reduced statistical power, increasing the risk of false-negative findings and limiting the generalizability of the results. In addition, the relatively small sample size and inclusion of multiple variables in multivariable models may increase the risk of overfitting and limit the robustness of the reported odds ratios and ROC analyses. Therefore, these findings should be considered exploratory.

It should be noted that ICU length of stay may be influenced by both cardiac and non-cardiac factors.

Differences in sampling timing may also have contributed to the lack of observed associations, as PGC-1α was measured in the early perioperative period, whereas hs-cTnT levels typically peaked later, postoperatively. This limitation should be addressed in future studies with larger sample sizes and serial measurements. Serial blood sampling was not performed to monitor the dynamic course of PGC-1α. This constraint limits a comprehensive interpretation of the biomarker’s continuous temporal kinetics.

Although MIRI is associated with significant morbidity and mortality, there is no consensus biomarker that can reliably assess myocardial injury. Although PGC-1α has been investigated in the context of cardiac energy metabolism, its underlying biochemical mechanisms remain incompletely understood, which also represents a limitation of this study.

Another important limitation is that, owing to the rapid progression of myocardial injury and reperfusion, obtaining adequately timed biomarker measurements is challenging, together with the lack of external validation.

### Contributions of the study to the literature and clinical practice

The value of this study is the combination of biochemical and cardiological data to examine PGC-1α during myocardial ischemia–reperfusion injury. These findings improve the understanding of PGC-1α’s clinical role and offer a foundation for future investigations.

Clinically, hs-cTnT and lactate levels remain the most reliable markers of acute myocardial injury and tissue hypoxia. In contrast, PGC-1α appears to be more strongly associated with metabolic adaptation and mitochondrial responses than with direct ischemic damage. Therefore, PGC-1α may have potential value as a complementary metabolic biomarker, particularly in patients with diabetes, and in predicting ICU-related outcomes.

## Conclusion

In conclusion, this study suggests that PGC-1α is not a marker of the duration of acute myocardial ischemia in patients undergoing coronary artery bypass grafting. However, the observed associations between PGC-1α levels, diabetes mellitus, and ICU duration indicate that this molecule may be more closely related to metabolic status and cellular adaptive processes than acute myocardial injury. These findings imply that PGC-1α may reflect mitochondrial and metabolic responses in myocardial ischemia–reperfusion injury rather than serving as a direct marker of myocardial damage. Further large-scale mechanistic studies are required to clarify the potential clinical utility of PGC-1α.

## Supplementary Information

Supplementary Material 1.

## Data Availability

The data that support the findings of this study are not openly available due to reasons of sensitivity and are available from the corresponding author upon reasonable request.
