# Supplementary figures and images for: Association of the mitochondrial regulator PGC-1α with diabetes mellitus and myocardial ischemia–reperfusion injury in coronary artery bypass grafting
Source: BMC Cardiovasc Disord. 2026 Jun 19;26:568. doi: 10.1186/s12872-026-06151-7 (PMC13339688; doi:10.1186/s12872-026-06151-7)

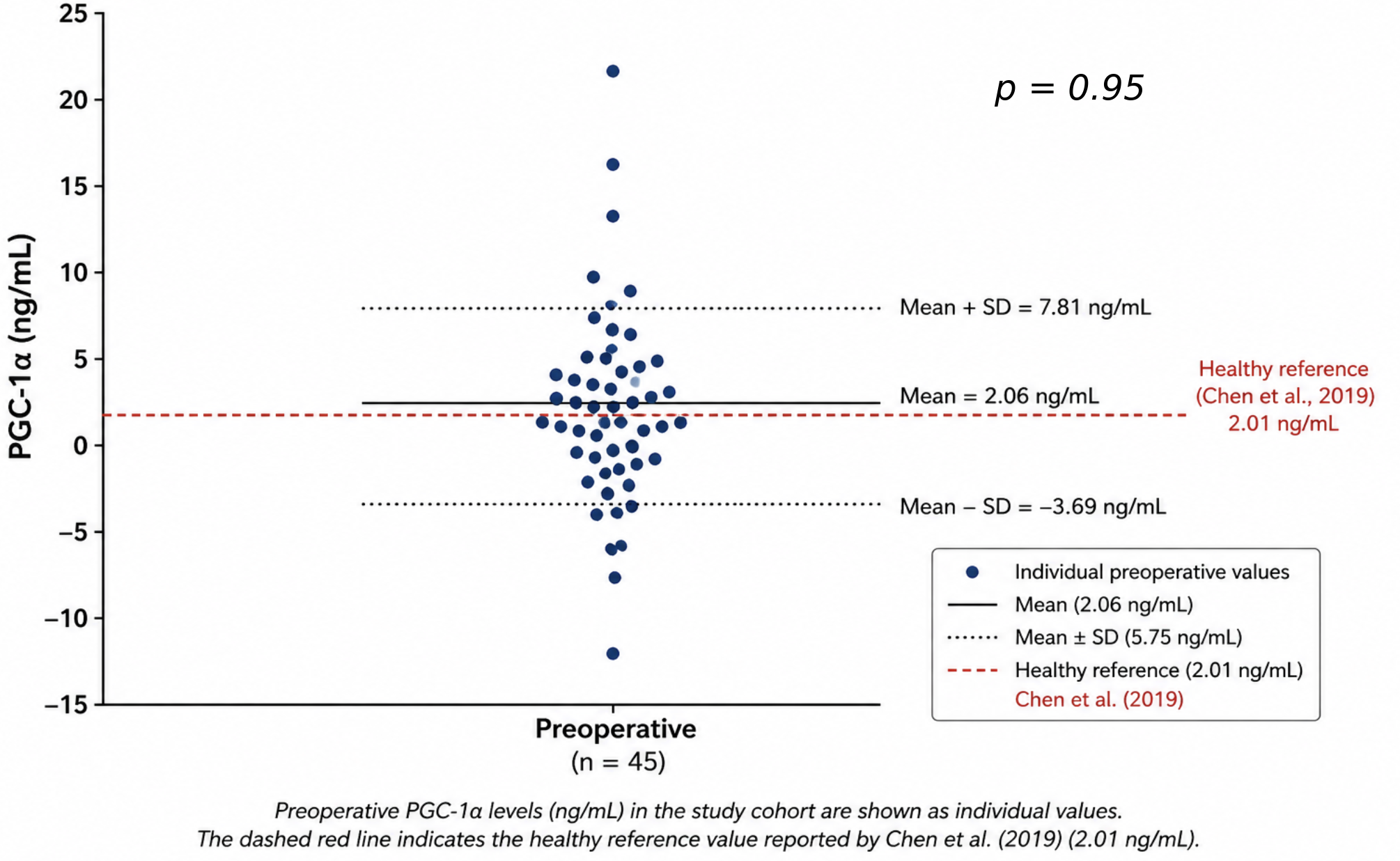

Supplement: Supplementary file 1 — Supplementary Material 1. [file 12872_2026_6151_MOESM1_ESM.docx]
